# Interventions to Promote Communication in Minimally Verbal Autistic Children: A Systematic Review

**DOI:** 10.3390/medsci14030388

**Published:** 2026-07-13

**Authors:** Roberta Privitera, Adriana Piccolo, Carmela De Domenico, Giulia Leonardi, Angelo Alito, Angelo Quartarone, Francesca Cucinotta, Marcella Di Cara

**Affiliations:** 1 IRCCS Centro Neurolesi Bonino-Pulejo, S.S. 113 Via Palermo, C. da Casazza, 98124 Messina, Italy; roberta.privitera@studio.unibo.it (R.P.); adriana.piccolo@irccsme.it (A.P.); carmela.dedomenico@irccsme.it (C.D.D.); angelo.quartarone@irccsme.it (A.Q.); marcella.dicara@irccsme.it (M.D.C.); 2 Department of Physical and Rehabilitation Medicine, University Hospital “G. Martino”, 98124 Messina, Italy; giulia.leonardi@polime.it; 3 Department of Biomedical, Dental Sciences and Morphological and Functional Images, University of Messina, 98125 Messina, Italy; alitoa@unime.it

**Keywords:** minimally verbal, communication, interventions, autism spectrum disorder, systematic review, rehabilitation

## Abstract

Background/Objectives: Minimally verbal autistic children face profound communication challenges that significantly affect daily functioning, including social interaction, behavioral regulation, and overall well-being. Identifying effective interventions is a key clinical-educational priority. This systematic review examines communication-focused interventions for these children, including both spoken language approaches and augmentative and alternative communication (AAC) strategies. Methods: The review was registered in PROSPERO and conducted following PRISMA-S guidelines. Four electronic databases were searched, and 18 studies met the inclusion criteria. Due to heterogeneity in study designs and outcome measures, a narrative synthesis was conducted, with subgroup analyses exploring sample characteristics, intervention types, targeted outcomes, and reported effects. Methodological quality of randomized controlled trials was assessed using the Cochrane Risk of Bias tool. Results: Interventions were implemented across clinical, educational, and home settings, using strategies such as Joint Attention, Symbolic Play, and Emotion Regulation, often combined with AAC technologies. Most included studies reported improvements in at least one communication outcome, although findings were heterogeneous and several studies reported mixed, subgroup-specific, or non-significant effects. The most consistent improvements were observed in spontaneous communication, particularly in interventions integrating developmental approaches with technological support. Conclusions: These findings highlight the value of individualized interventions. Future research should establish standardized definitions of “minimally verbal”, assess long-term outcomes, and adopt broader measures capturing both language and social-emotional development to enhance quality of life and inclusion.

## 1. Introduction

Autism Spectrum Disorder (ASD) is a neurodevelopmental condition characterized by persistent difficulties in social communication and restricted, repetitive behaviors [1,2]. Developmental differences in cognition and language are associated features of the autism phenotype [1]. Current estimates note that about 20–30% of individuals on the autism spectrum remain minimally verbal (MV), meaning they use few or no functional spoken words in daily communication [3,4,5,6]. These data highlight the urgent need for targeted and early interventions. However, identifying MV individuals and selecting appropriate treatments remains challenging, partly due to the absence of standardized and widely accepted diagnostic criteria for MV ability [7]. This lack of consensus not only complicates early identification in clinical settings but also hinders the reliable characterization and comparison of MV profiles across studies, limiting the generalizability of research findings. Indeed, MV children often present with complex and heterogeneous developmental profiles, including co-occurring cognitive and behavioral challenges [8]. Their difficulties extend beyond spoken language, affecting nonverbal communication channels such as joint attention, gestures, and symbolic play [9]. These impairments can hinder inclusion, social-emotional development, and autonomy [10,11,12].

In recent years, scientific and policy attention on interventions for MV autistic children has grown significantly. Since 2010, when the National Institutes of Health identified this population as a research priority, several strategic documents, most notably the Interagency Autism Coordinating Committee Strategic Plan, have underscored the need for targeted support. The latest update [13] emphasizes the urgent need to develop stratification and prognostic biomarkers that can identify which autistic children are most likely to benefit from specific interventions, as well as biomarkers and interventions that promote language development during early sensitive developmental periods.

In response, a variety of interventions have been developed to support communication in this population. These include behavioral strategies, developmental approaches, and augmentative and alternative communication (AAC) systems [14], which aim either to foster spoken language or to support multimodal communication. However, the current literature is highly fragmented, with inconsistent definitions, heterogeneous methods, and limited generalizability [4,15]. Several reviews have previously examined interventions for minimally verbal autistic children, although with different scopes and methodological criteria. Brignell et al. [4] focused exclusively on randomized controlled trials of communication-focused interventions in minimally verbal children, resulting in a highly selective evidence base. Koegel et al. [15] focused on interventions aimed at increasing verbal speech production. More recently, Pope et al. [16] specifically investigated the effects of Naturalistic Developmental Behavioral Interventions (NDBIs) with and without AAC, restricting inclusion to interventions meeting specific NDBI criteria and to studies with experimental designs. While these highly selective criteria are valuable for establishing evidence of intervention efficacy under controlled conditions, they often exclude a substantial body of practice-based evidence, observational studies, and innovative pilot interventions that better reflect the heterogeneity of real-world clinical settings.

In light of these methodological and conceptual constraints, there is a critical need for a comprehensive synthesis that maps the full spectrum of available evidence beyond the restrictive methodological inclusion criteria adopted in previous reviews. Such a synthesis can provide clinicians and researchers with a broader understanding of currently available intervention strategies. The current review adopts a broader and more inclusive perspective to bridge this gap. Unlike previous reviews, the present review is not limited to evidence derived from highly controlled intervention studies. Instead, it provides a comprehensive overview of the diversity of communication interventions currently available for minimally verbal autistic children, thereby informing both future research priorities and clinical decision-making. Specifically, this review includes different intervention approaches targeting communication in minimally verbal autistic children and considers a wider range of study designs.

By integrating findings from randomized controlled trials (RCT), observational studies, and pilot projects, this review aims to identify promising intervention strategies that might be overlooked in narrower syntheses, to capture real-world clinical utility, and to highlight areas where further research is needed. Supporting all forms of communication in MV autistic children is not only a clinical necessity but also an ethical imperative, crucial for fostering inclusion, safeguarding dignity, and improving overall quality of life.

## 2. Methods

### 2.1. Search Strategy and Data Sources

This systematic review was registered in PROSPERO database (CRD42025642752) and conducted without substantive deviations from the registered protocol, in accordance with PRISMA-S guidelines [17,18] (see the Supplementary Materials Table S1). A comprehensive search was performed across four databases (Embase, Web of Science, Cochrane Library, and PubMed) on 28 February 2025, with no restrictions on publication date. The search strategy combined the term “autism” with “minimally verbal” or “minimally speaking” (see Supplementary Materials). Reference lists of included studies and relevant reviews were also screened to identify additional articles. The complete search strategies for each database are reported in Supplementary Materials.

### 2.2. Eligibility Criteria

Studies were eligible if they included participants younger than 18 years with a formal diagnosis of ASD and minimally verbal status, evaluated communication-focused interventions, and used randomized, non-randomized, pilot, or observational study designs. Only peer-reviewed articles published in English or Italian (or with an available English translation) were considered. Case reports, reviews, editorials, conference abstracts, and studies not meeting the predefined eligibility criteria were excluded. Full eligibility criteria are reported in the Supplementary Materials.

### 2.3. Study Selection and Data Extraction

Two authors independently screened titles, abstracts, and full-text articles for eligibility. Disagreements were resolved through discussion and, when necessary, by consultation with a third author. Two authors independently extracted data using a predefined coding framework covering study characteristics, participant demographics, intervention features, and outcome measures. Any discrepancies were resolved by consensus.

### 2.4. Strategy for Data Synthesis

Due to heterogeneity in methods and outcomes, a narrative synthesis was conducted. Sub-analyses examined: (a) sample characteristics (age, gender, ethnicity, autism severity, Nonverbal Intellectual Quotient (NVIQ), definitions of MV status, and comorbidities); (b) intervention technical features (intensity, frequency, duration); (c) outcome measures; and (d) effects by intervention type.

Interventions were categorized by theoretical orientation (e.g., NDBI, AAC-based, parent- or peer-mediated). Findings were summarized descriptively, highlighting patterns of improvement and contextual differences across studies.

### 2.5. Quality Assessment

RCTs were evaluated using the Cochrane Risk of Bias tool [19] across seven domains, while non-randomized studies were assessed via the ROBINS-I tool [20]. To allow cross-design comparability within a single quality framework, domain-level assessments from both tools were subsequently mapped onto Agency for Healthcare Research and Quality (AHRQ) standards [21], yielding three summary categories: “good quality” (low risk of bias across all or nearly all domains), “fair quality” (some concerns or moderate risk in one or more domains, unlikely to substantially affect outcome validity), and “poor quality” (serious or critical risk of bias in one or more key domains). This conversion was adopted because RoB 2 and ROBINS-I produce domain-specific judgements that are not directly comparable across study designs; the AHRQ framework provides a common summary scale that facilitates interpretation across mixed-design systematic reviews [21]. The mapping followed the following correspondence criteria: for RCTs, a “low risk” rating across all Cochrane Risk of Bias tool domains corresponded to AHRQ “good”; “some concerns” in one or more domains to “fair”; and “high risk” in any domain to “poor.” For non-randomized studies, a ROBINS-I overall rating of “low” or “moderate” risk mapped to AHRQ “good” or “fair” respectively, while “serious” or “critical” risk mapped to “poor.” This approach has been applied in prior systematic reviews combining RCT and non-randomized evidence [11,22,23]. Borderline cases were resolved by consensus between two independent reviewers, with discrepancies arbitrated by a third.

The full domain-level Cochrane Risk of Bias tool and ROBINS-I assessments, alongside the corresponding AHRQ summary ratings, are reported in Supplementary Tables S5 and S6, allowing independent verification of the mapping.

## 3. Results

The search identified 1088 records (PubMed n.261; Embase n.336; Cochrane Library n.71; Web of Science n.420). After removing 659 duplicates, 429 articles were screened and 385 excluded (population n.21; publication type n.71; study design n.39; background/non-original n.252; outcome n.1; language n.1).

**Figure 1:**
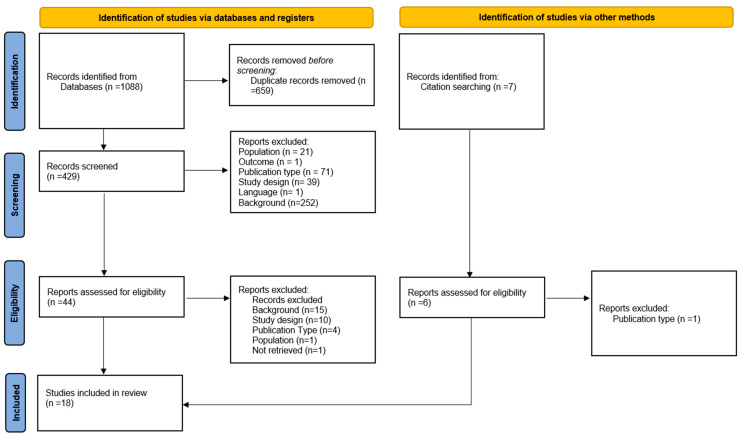
PRISMA flow diagram for literature search strategy. The figure outlines the search and review process with the total number of articles included and excluded in this review.

Forty-four full texts were assessed, with 30 excluded (background n.15; study design n.10; publication type n.4; population n.1). Seven additional records were identified through citation searching (six included, one excluded), and one eligible article was not retrieved. In total, 18 studies were included (Figure 1).

The included studies were published between 1988 and 2023 and were mainly conducted in the United States (n.13; 72.2%), with others from Asia (n.2; 11.1%), Europe (n.1; 5.5%), and US–Europe collaborations (n.2; 11.1%). Fifteen were RCTs (including three pilot and two SMART designs), two used within-subject designs, and one was quasi-experimental (Supplementary Table S2).

### 3.1. Sample Characteristics

The sample ranged from 10 to 164 participants (M = 49.3 ± 37.86), aged 2.4–11.99 years old (M = 5.87; SD = 1.39). Overall, 13 studies (72.2%) reported NVIQ: Eight (44.4%) provided standardized scores (means ≈ 22.75–71.2; overall ≈ 47), three (18.7%) reported mental age (≈ 4.0 years; not directly convertible), and two used categorical levels (“low”/“high”) via the Mullen Scales of Early Learning (MSEL) [24]. Ethnicity was reported in 10/18 studies (2 incomplete); in the remaining sample (n.507), 169 were White, 73 Hispanic/Latino, 60 Non-White, 6 Black, 18 Asian, 12 Asian American, 26 African American, 131 Non-Hispanic, and 12 Other. Gender was reported in 15 studies (625 males, 122 females); excluding inconsistent data, 13 studies showed M:F = 5.12:1. All participants had confirmed ASD and were MV; diagnoses were established via the DSM IV/5 [1,25] or ICD 9/10 [26,27] except in one study, which was typically supported by the Autism Diagnostic Observation Schedule (ADOS-G/ADOS-2; n.13; 81.3%) [28,29], Autism Diagnostic Interview–Revised (ADI-R; 2; 12.5%) [30], and Childhood Autism Rating Scale (CARS; 2; 12.5%) [31], and less frequently Gilliam Autism Diagnostic Scale Second Edition (GARS-2) [32], Social Communication Questionnaire (SCQ) [33], and Social Responsiveness Scale (SRS) [34]. Five studies (27.5%) reported moderate–severe ASD using standardized severity measures. MV definition varied, mainly based on expressive vocabulary thresholds: ≤20 words (seven studies; 38.8%), <30 (4; 22.2%), ≤25 (3; 16.6%), or other cut-offs (<50, 15, or 10 words; 22.2%), all referring to spontaneous, functional speech. Assessment methods included parent reports (nine studies; 50%), Naturalistic Language Samples (NLS) (6; 33.3%), structured observations/interaction coding (4; 22.2%) and standardized tools: ADOS (n.4; 22.2%) [28,29], MSEL (3; 16.6%) [24], Vineland Adaptive Behavior Scales (VABS; 2; 11.1%) [35], Communication and Symbolic Behavior Scales (CSBS;2; 11.1%) [36], MacArthur-Bates Communicative Development Inventory (CDI; n.1) [37], and Peabody Picture Vocabulary Test (PPVT, n.1) [38]. Only two studies reported comorbidities (intellectual disability, cerebral palsy, and seizures). Detailed demographic and diagnostic data are provided in Supplementary Table S3.

### 3.2. Intervention Technical Standards

The 18 included studies tested diverse interventions with varied aims and methods. Most were in clinical settings (6; 33.3%), followed by schools (5; 27.7%) and clinical–home contexts (3; 16.6%). One was fully remote during COVID-19, while 3 (16.6%) did not specify the setting. Intervention parameters varied widely: number of sessions ranged from 1–24 (4; 22.2%), 25–45 (4; 22.2%), 46–70 (4; 22.2%), to >70 (3; 16.6%); 3 (16.6%) did not report session numbers. Weekly frequency varied: 1/week (1 study), 2/week (2; 11.1%), 2–3/week (6; 33.3%), 3–4/week (2; 11.1%), ≥5/week (2; 11.1%); 3 were unspecified (16.6%) and 1 excluded (single session). Session length ranged from <30 min (2; 11.1%) and 30–44 (2; 11.1%) to 45–59 (3; 16.6%), most commonly 60–69 (6; 33.3%), with single cases at 90 and 120 min; 3 unspecified (16.6%). Intervention duration was ≤8 weeks (2; 11.1%), 9–15 (5; 27.7%), 16–23 (3; 16.6%), and ≥24 (5; 27.7%); 2 unspecified (11.1%) and 1 excluded (single session). Detailed technical parameters are reported in Supplementary Table S4.

### 3.3. Outcome Measures and Targeted Results

The included studies used a wide range of instruments to assess communication and language outcomes, indicating substantial methodological heterogeneity. Both standardized and observational tools were employed. The most frequent measures were the MacArthur CDI (n.4; 22.2%), MSEL (n.3; 16.6%), and VABS (n.3; 16.6%). Other instruments included PPVT, Reynell Developmental Language Scales (RDLS), Preschool Language Scale, and Expressive One-Word Picture Vocabulary Test (each n.2; 11.1%) [39,40,41]. Observational approaches were also common, including NLS video coding, Structured Play Assessment, and Early Social Communication Scales (each n.4; 22.2%), as well as parent/peer/teacher–child interaction measures (n.9; 50%) and intervention-session coding (n.2; 11.1%). Sixteen studies assessed expressive and receptive language, primarily via standardized or structured tools (10/16; 62.5%), followed by video-based coding (5/16; 31.2%), parent questionnaires (4/16; 25%), and semi-structured interviews (3/16; 18.7%). Expressive language refers to conveying meaning through speech, gestures, or signs, whereas receptive language involves understanding communication through auditory and visual input [42].

Spontaneous communication refers to unprompted communicative acts [43] and was examined in 11 studies (61.1%), mainly using video recordings (11/18), sometimes supported by questionnaires or structured tools (each 2/11; 18.2%). Social communication reflects context-appropriate verbal and nonverbal interaction [44] and was investigated in 10 studies (55.5%), using structured observations (5/10; 50%), interviews (3/10; 30%), questionnaires (2/10; 20%), video recordings (2/10; 20%), and one standardized test (10%). Play skills were assessed in 6 studies, mostly via video coding (5/6), with 1 study combining standardized and parent-report measures (1/6; 16.6%). Play skills involve functional and symbolic object use, peer interaction, and support cognitive, social, and language development [45].

Two studies also examined speech sound accuracy (video-based) [46] and phonological/phonemic awareness (non-standardized parent questionnaire) [47]. A detailed overview of outcome measures is provided in Table 1.

### 3.4. Naturalistic Developmental Behavioral Interventions

Six studies (33.3%) implemented NDBIs, integrating developmental and applied behavior analysis principles to enhance communication and social interaction within child-led, play-based contexts. Most interventions combined Joint Attention, Symbolic Play, Engagement and Regulation (JASPER) with Enhanced Milieu Teaching (EMT), sometimes augmented with Speech-Generating Devices (SGDs) to support expressive language in MV autistic children.

In Almirall et al. [48], a two-stage adaptive design compared JASP+EMT alone versus with SGDs over 24 weeks (2 sessions/week). JASP targeted joint attention, engagement, and symbolic play, while EMT focused on modeling and prompting language in natural routines. Non-responders received intensified or augmented treatment. Starting with SGDs yielded greater gains in spontaneous communication and social initiations.

Similarly, Chang et al. [49] applied JASP+EMT (±SGDs) over six months (2 weekly 1 h sessions), emphasizing symbolic and functional play. No between-group differences emerged for play, but improvements in symbolic play correlated with expressive language gains, supporting their developmental link.

In the preschool study by Goods et al. [50], JASPER supplemented existing ABA programs (2 × 30 min/week for 12 weeks). Structured play routines promoted joint attention and gestures without SGDs. Children showed increased play diversity and communicative initiations, with classroom generalization.

Kasari et al. [51] used a similar SMART design with JASP+EMT (2 × 1 h/week for 12 weeks), adapting intensity or adding SGDs for slow responders and later involving parents. Early SGDs plus intensified support produced the strongest gains in spontaneous utterances and novel word use, highlighting the value of early multimodal input.

In a parent-mediated model, Siller et al. [52] evaluated the Focused Playtime Intervention (FPI) across 12 home sessions, enhancing parental responsivity through modeling, feedback, and video review. Improvements in caregiver behavior were associated with expressive language gains, particularly in children with lower baseline skills.

Finally, the PRISM model by Barrett et al. [53], combining parent-mediated and clinician-delivered Pivotal Response Treatment (PRT), targeted social motivation through natural reinforcers and affective prompts during play. Over six months, it improved social responsiveness and language complexity, especially in MV children.

### 3.5. Structured Behavioral Approaches (DTT and Variants)

Three studies examined both structured and naturalistic interventions to support spoken language in MV autistic children. Discrete Trial Training (DTT), a highly structured ABA-based method using repeated stimulus–response–consequence cycles, was a core component across studies.

In Hampton et al. [54], DTT was embedded within a multi-component intervention combining JASP+EMT+SGDs and parent training. It targeted foundational skills (imitation, joint attention, basic SGD use) and led to significant improvements in joint attention post-intervention and in social communication at 4-month follow-up.

Kasari et al. [55] directly compared DTT with JASPER, a NDBI focused on symbolic play, engagement, and regulation. Both approaches improved expressive language, but outcomes varied by baseline profiles: children with stronger receptive language and joint attention benefited more from JASPER, whereas those with broader developmental delays showed greater gains with DTT at follow-up.

Finally, Paul et al. [56] evaluated Rapid Motor Imitation Antecedent (RMIA), an enhanced DTT approach using motor imitation sequences to increase vocal imitation. RMIA was compared to Milieu Communication Training (MCT), a naturalistic method based on modeling, time delay, and incidental teaching; both included parent responsivity training. While both groups showed similar gains in spoken words, treatment response was moderated by child characteristics: RMIA was more effective for children with lower receptive language, whereas MCT was more beneficial for those with stronger joint attention and comprehension.

### 3.6. Speech-, Music-, and Imitation-Based Interventions

Speech-, music-, and imitation-based interventions represent innovative approaches to promote verbal production in MV or nonverbal autistic children by leveraging rhythm, melody, and motor imitation.

Auditory-Motor Mapping Training (AMMT), described by Chenausky et al. [46], combines intoned bi-syllabic speech with bilateral rhythmic drumming on tuned instruments, based on the premise that simultaneous auditory–motor activation strengthens speech-related cortical networks. Results showed significant improvements in phonemic accuracy and generalization to untrained items.

Similarly, Sandiford et al. [57] evaluated the Melodic-Based Communication Therapy (MBCT), which pairs target words with specific melodies and rhythmic clapping. In this randomized pilot study, MBCT elicited more imitative verbal attempts and greater parent-reported novel word use at home compared to standard speech-language therapy.

In a RCT, Yoder and Layton [58] compared four input modalities (speech only, sign only, simultaneous speech + sign, alternating speech + sign) in 60 MV autistic children. Modalities including spoken input produced greater spontaneous spoken word use than sign alone, particularly in children with stronger baseline verbal imitation.

Overall, these findings suggest that melodic, rhythmic, and imitation-based interventions can enhance speech development in MV children, with pre-treatment imitation ability emerging as a key moderator of outcomes.

### 3.7. AAC Interventions

Three studies examined AAC-based interventions targeting expressive communication in young autistic children with limited speech. One randomized trial [59] compared PRT, a verbally mediated approach, with the Picture Exchange Communication System (PECS), a pictorial AAC system, both delivered intensively across home and clinical settings with parent training. No significant group differences emerged in standardized spoken language outcomes; however, most children in the PECS group progressed to higher system phases, indicating more complex functional communication via picture exchange. Parental satisfaction was high for both interventions, although PECS was reported as more demanding to implement.

Thiemann-Bourque et al. [60] evaluated a peer-mediated intervention using a tablet-based SGD, where typically developing peers were trained as communication partners within natural preschool activities. Children in the intervention group showed significant improvements in communication rate and reciprocity compared to controls, with gains generalizing across settings and maintained over time. Implementation fidelity was high, supported by structured peer training.

In a pragmatic group randomized trial, Howlin et al. [61] assessed PECS training for teachers, delivered through a two-day workshop with ongoing support and implemented over five months. The intervention led to significant increases in picture-based communication initiations, although spoken language gains were limited, demonstrating the feasibility of teacher-delivered AAC in educational settings.

Overall, these findings highlight the effectiveness of AAC interventions, both pictorial and speech-generating, in enhancing expressive communication in MV autistic children. Embedding AAC within multimodal, socially interactive contexts, including peer- and teacher-mediated models, appears to support both communication and social engagement.

### 3.8. Experimental and Hybrid Interventions

Three studies implemented innovative, multimodal approaches targeting early lexical development in MV autistic children. Abdi et al. [62] developed a combined intervention grounded in behaviorism, schema theory, socio-cultural theory, and event representation theory. Across 16 sessions, children and caregivers engaged in structured routines, symbolic exercises, and parent-mediated interactions to scaffold vocabulary through meaningful, repeated participation in daily activities. The approach integrated contextualized and decontextualized learning, supporting a transition from action-based to symbolic communication. Significant gains in expressive and receptive vocabulary were observed and maintained at follow-up.

Clark et al. [47] examined a computer-based mutual exclusivity task with orthographic support. Children learned novel word–object pairings with or without written input; recall was higher with orthographic cues, particularly in those with lower expressive language, highlighting the value of visually supported input in remote contexts.

In a school-based peer intervention [63], dyads of MV students (aged 8–16) participated in a 15-week program targeting either nonverbal social conversation or collaboration skills through structured activities (e.g., gesture-based games, cooperative tasks). Both conditions improved spontaneous peer interaction, while the conversation-focused group also showed gains in teacher-rated socialization and executive functioning.

### 3.9. Synthesis of Main Effects

**Figure 2:**
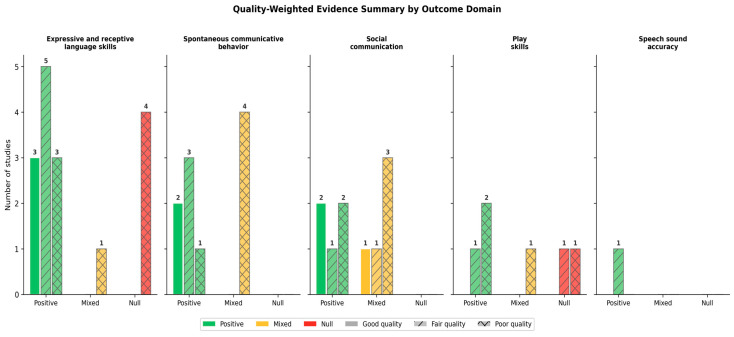
Quality-weighted evidence summary by outcome domain. For each outcome, bars represent the number of studies reporting positive, mixed, or null results (*x*-axis), grouped by AHRQ rating. Bar color indicates result direction (green = positive; yellow = mixed; red = null); hatching indicates study quality (solid = Good; diagonal = Fair; cross = Poor). Numbers above bars indicate the number of studies in each category.

The findings indicate a mixed but generally favorable pattern of effects (Figure 2). For expressive and receptive language, 10/16 studies (62.5%) reported significant or sustained improvements, while 6/16 (37.5%) found no effects. Standardized tests most consistently detected gains: Kasari et al. [55] and Schreibman & Stahmer [59] showed broad improvements, Thiemann-Bourque et al. [60] stronger expressive effects, and Hampton et al. [54] only short-term gains.

Parent questionnaires captured vocabulary growth, though sustained effects were observed only for RMIA in Paul et al. [56], a pattern confirmed by interviews. Video-based analyses were more variable: Barrett et al. [53] and Sandiford et al. [57] reported improvements in social responsiveness, utterance length, imitation, and novel words; Kasari et al. [55] identified condition-specific effects, Hampton et al. [54] increased caregiver–child exchanges, whereas Paul et al. [56] found no effects. Yoder et al. [58] further showed that imitation, age, and IQ predicted outcomes, with poorer performance in the “sign alone” condition.

Spontaneous communication improved in 8/11 studies (72.7%), with one partial [56], one trend-level [54], and one null result [55]. Evidence was mainly based on video observations, consistently showing gains in responsiveness, conversational quality, and spontaneous utterances, as well as increased gestures, eye contact, and balanced exchanges—particularly in MV children and collaborative contexts. Standardized measures were less frequent but confirmed sustained gains in Paul et al. [56], while Howlin et al. [61] observed delayed improvements at 10 months. Questionnaire data indicated partial attainment of functional language milestones [56] and perceived improvements over time [60].

Play skills showed more variable results: 3 studies reported significant improvements, 2 mixed/null effects, and 1 partial evidence depending on measures. Video-based analyses (5/18; 27.7%) showed increased play diversity [50], collaborative engagement [63], and broader gains across groups with additional follow-up advantages for DTT [55]. In contrast, Almirall et al. [48] and Chang et al. [49] found no between-group differences. Standardized measures indicated sustained gains, while questionnaires suggested limited functional outcomes [56].

Evidence for social communication was strongest in observational studies. Almirall et al. [48] and Kasari et al. [55] reported improved joint attention with SGD/JASPER, while behavioral regulation improved across groups. Howlin et al. [61] found delayed but significant gains, though still within the clinical range. Siller et al. [52] showed improved maternal synchronization, and Thiemann-Bourque et al. [60] reported sustained gains in communicative reciprocity. Other findings were mixed: Goods et al. [50] found reduced unengaged time but limited joint attention changes; Paul et al. [56] reported partial effects; Schreibman et al. [59] showed improvement over time; and Bauminger-Zviely et al. [63] found minimal effects. For additional outcomes, Chenausky et al. [46] reported significant gains in speech sound accuracy, whereas Clark et al. [47] found no effects on phonological awareness.

Overall, the evidence supports robust effects on expressive language and spontaneous communication, more variable outcomes for play skills, and less consistent results for social communication and phonological awareness. Detailed outcomes are reported in Table 1.

### 3.10. Quality Assessment

**Figure 3:**
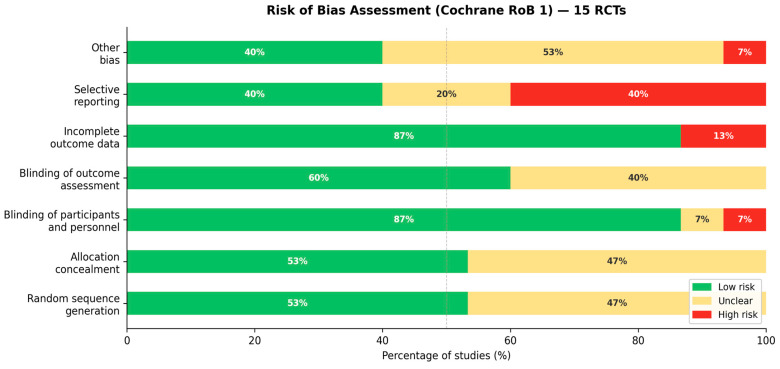
Weighted bar chart showing the distribution of risk-of-bias judgements across the seven domains of the Cochrane Risk of Bias tool for the 15 included RCTs. Values represent the percentage of studies rated low risk, unclear, or high risk per domain.

Of the 15 RCTs assessed using the Cochrane Risk of Bias tool, three (20%) were rated ‘good quality’, five (33.3%) ‘fair quality’, and seven (46.7%) ‘poor quality’ according to AHRQ standards. The domain-level results are summarized in Figure 3 and detailed in Supplementary Table S5.

Random sequence generation was adequately described in eight studies (53.3%); seven studies (46.7%) were unclear, and no study was rated as high risk. Allocation concealment was specified in eight studies (53.3%; unclear in seven studies, 46.7%; no study rated high risk). Blinding of participants and personnel was at low risk in 13/15 (86.7%) studies, unclear in 1/15 (6.7%) and at high risk in 1/15 (6.7%). Blinding of outcome assessment was at low risk in nine studies (60.0%) and at unclear risk in six studies (40.0%). Incomplete outcome data were at low risk in 13/15 studies (86.7%) and at high risk in 2/15 studies (13.3%). Selective outcome reporting was the most problematic domain, with a high risk rating in six studies (40%), a low-risk rating in six studies (40%), and an unclear rating in three studies (20%). Other sources of bias were unclear in eight studies (53.3%), low risk in six studies (40.0%), and high risk in one study (6.7%). Only three RCTs (20.0%) were prospectively registered in a public registry, contrary to the 2010 CONSORT recommendations.

Of the three non-randomized studies assessed using ROBINS-I, two (66.7%) were rated ‘poor quality’ [56,62] and one (33.3%) ‘fair quality’ [47]. Confounding was the most consistently problematic domain, rated ‘critical’ in one study [62], ‘serious’ in one study [56], and ‘moderate’ in one study [47]. Selection of participants into the study was rated ‘serious’ in two studies (66.7%) and ‘moderate’ in one study (33.3%). Classification of intervention was consistently low risk across all three studies (100%). Missing data were well managed in two studies (66.7%; low risk) and of moderate quality in one study (33.3%). Measurement of outcomes and selection of reported results were rated as ‘serious’ or ‘moderate’ in two studies each. Only one study [47] achieved a ‘low-risk’ rating for both domains. Supplementary Tables S5 and S6 show the detailed characteristics of the included studies.

### 3.11. Summary of Main Findings

The main findings of the included studies can be summarized as follows:

○ Expressive and receptive language improved in most studies (10/16; 62.5%), particularly when assessed using standardized language measures.
○ Spontaneous communication showed the most consistent positive results (8/11; 72.7%), with improvements in communicative initiations, conversational exchanges, and social reciprocity.
○ Naturalistic Developmental Behavioral Interventions (NDBIs) and interventions incorporating Speech-Generating Devices (SGDs) were among the approaches most consistently associated with positive communication outcomes.
○ Play skills and social communication showed more variable findings across studies, reflecting substantial methodological heterogeneity.
○ Considerable variability was observed in intervention characteristics, outcome measures, and methodological quality, limiting direct comparison across studies and highlighting the need for greater standardization in future research.

**Table 1:** Outcome assessment and main results.

ID	Author	Measurement Instrument	Outcome Domain	Assessment Type	Main Results
1	Abdi S. et al., 2023 [62]	CDI-1	Expressive and receptive	Parent-reported measure	Significant improvements in expressive vocabulary and receptive language, maintained at follow-up.
2	Almirall D. et al., 2016 [48]	NLS-videocoding	Spontaneous Communicative B.	Observational measure	JASP+EMT+SGDs significantly increased spontaneous communicative utterances; no between-group differences in novel word acquisition.
		Structured Play Assessment	Play skills	Observational measure	No significant between-group differences in play; no significant change over time.
		Early Social Communication Scales	Social communication skills	Structured observational measure	SGDs improved joint attention; behavioral regulation improved similarly across all groups and was maintained over time.
3	Barrett, AC et al., 2020 [53]	Parent–child interaction	Spontaneous Communicative B.; Expressive and receptive	Observational measure	Intervention improved social responsiveness and utterance length. Baseline social responsiveness predicted later language outcomes.
4	Bauminger-Zviely, N. et al., 2020 [63]	Peers–child interaction	Spontaneous Communicative B., Play Skills	Observational measure	Collaboration group showed greater active participation and reduced passivity; no group differences in play skills.
		Social Conversation Scale	Spontaneous Communicative B.	Observational measure	Both groups improved conversation quality; collaboration group showed greater gains in gestures, eye contact, and peer relevance.
		VABS	Expressive and receptive; Social communication skills	Semi-structured interview	No significant improvement in communication. Socialization improved mainly in the conversation group.
5	Chang YC et al., 2018 [49]	PPVT-4	Expressive and receptive	standardized measure	Play skills were positively associated with expressive language.
		Structured Play Assessment	Play skills	Observational measure	Both groups improved play skills over six months.
6	Chenausky, K.V. et al., 2022 [46]	% Syllables Approximately Correct, % Consonants Correct, % Vowels Correct	Speech sound accuracy	Observational measure	AMMT produced greater improvements in speech sound accuracy than SRT.
7	Clark, G. T. et al., 2023 [47]	CDI-1	Expressive and receptive	Parent-reported measure	No significant effect of orthography during the learning task.
		checklist regarding their child’s literacy skills	Phonological and Phonemic Awareness	Non-standardized parent-reported measure	Orthography-present condition increased exposure to newly learned words at home.
8	Goods KS et al., 2013 [50]	RDLS	Expressive and receptive	standardized measure	No between-group differences in expressive or receptive language
		Structured Play Assessment	Play skills	Observational measure	Intervention increased play diversity.
		Peers/Teachers-child interaction	Spontaneous Communicative B., Social communication skills	Observational measure	Intervention reduced unengaged behavior and increased requesting gestures.
		Early Social Communication Scales	Social communication skills	Structured observational measure	No significant improvements in joint attention or behavioral regulation.
9	Hampton, L.H. et al., 2020 [54]	PLS-5	Expressive and receptive	standardized measure	Small post-intervention advantage in expressive language; effects were not maintained at follow-up.
		NLS-videocoding	Spontaneous Communicative B.	Observational measure	No significant between-group differences
		Parent–child interaction	Spontaneous Communicative B.; Expressive and receptive	Observational measure	Intervention increased caregiver–child social communicative exchanges at follow-up
		Early Social Communication Scales	Social communication skills	Structured observational measure	Joint attention improved immediately after treatment but not at follow-up.
10	Howlin et al., 2007 [61]	The Expressive One Word Picture Vocabulary Test	Expressive and receptive	standardized measure	No significant treatment effects on standardized language measures.
		British Picture Vocabulary Scales	Expressive and receptive	standardized measure	No significant treatment effects on standardized language measures.
		Peers–child interaction	Spontaneous Communicative B.	Observational measure	Spontaneous communicative initiations increased immediately after treatment but were not maintained.
		ADOS G	Spontaneous Communicative B., Social communication skills	Structured observational measure	Delayed improvement observed only at 10-month follow-up.
11	Kasari C. et al., 2014 [51]	PPVT-4	Expressive and receptive	standardized measure	JASP+EMT+SGDs produced better communication outcomes than JASP+EMT.
		NLS-videocoding	Spontaneous Communicative B.	Observational measure	Greater gains in spontaneous communication with JASP+EMT+SGDs, maintained at follow-up.
		Intervention Session Transcripts	Spontaneous Communicative B.	Observational measure	Best outcomes achieved with early SGD introduction and adaptive treatment intensification.
12	Kasari, C. et al., 2023 [55]	RDLS	Expressive and receptive	standardized measure	Both groups improved language; no significant between-group differences.
		NLS-videocoding	Spontaneous Communicative B.	Observational measure	Both groups improved in SCU and NDWR; small advantage for DTT in NDWR at exit
		Structured Play Assessment	Play skills	Observational measure	Both groups improved play; DTT showed greater long-term gains in play level.
		Parent–child interaction	Spontaneous Communicative B.; Expressive and receptive	Observational measure	Both groups improved language; DTT showed greater expressive and receptive gains at selected assessments.
		Early Social Communication Scales	Social communication skills	Structured observational measure	JASPER improved joint attention; both groups improved behavioral regulation.
13	Paul R. et al., 2013 [56]	CDI-1	Expressive and receptive	Parent-reported measure	Both interventions increased spoken vocabulary, with gains maintained at follow-up.
		Parent–child interaction	Spontaneous Communicative B.; Expressive and receptive	Observational measure	Parent responsiveness did not moderate treatment response.
		VABS	Expressive and receptive; Social communication skills	Semi-structured interview	RMIA improved expressive language; MCT showed no significant changes.
		CBCS—Developmental Profile	Spontaneous Communicative B.; Social communication skills; Play Skill	Standardized measure	Both interventions increased spontaneous verbal production.
		Caregiver Questionnaire of the CSBS	Spontaneous Communicative B.; Social communication skills; Play Skill	Parent-reported measure	Functional language milestones achieved in both groups; no significant between-group differences.
14	Sandiford, GA et al., 2013 [57]	Intervention Session Interaction	Spontaneous Communicative B.; Expressive and receptive	Observational measure	Both groups improved; MBCT produced earlier gains and greater vocabulary growth.
15	Schreibman, LE et al., 2014 [59]	MSEL	Expressive and receptive	Standardized measure	Significant improvement over time for both groups, without significant difference between interventions.
		Expressive One-Word Picture Vocabulary Test-Revised	Expressive and receptive	Standardized measure	Significant improvement over time
		CDI-1	Expressive and receptive	Parent-reported measure	Significant increase in words produced
		VABS	Expressive and receptive; Social communication skills	Semi-structured interview	Significant improvement over time
16	Siller et al., 2013 [52]	MSEL	Expressive and receptive	Standardized measure	No significant main effect of treatment on expressive language gains
		Therapist-child interaction	Social communication skills	Observational measure	Significant main effect of treatment on maternal synchronization gains from baseline to exit
17	Thiemann-Bourque K. et al., 2018 [60]	PLS-5	Expressive and receptive	Standardized measure	Both groups improved significantly in expressive and receptive language.
		Peers–child interaction	Spontaneous Communicative B.; Social communication skills	Observational measure	Intervention increased communication rates, interaction quality, and generalization across settings.
		MSEL	Expressive and receptive	Standardized measure	Both groups improved significantly; the treatment group showed significantly greater gains in expressive language
		SIRS	Spontaneous Communicative B.	Parent/Teacher-reported measure	Teachers and parents reported significant improvements over time
18	Yoder PJ et al., 1988 [58]	Elicited Verbal Imitation Measure	Expressive	Structured non-standardized measure	Verbal imitation predicted spontaneous speech. Sign-alone training resulted in fewer spontaneous words; age and IQ predicted better spoken vocabulary.

ADOS-G: Autism Diagnostic Observation Schedule-Generic; AMMT:Auditory-Motor Mapping Training; CBCS:Communication and Symbolic Behavior Scales; CDI-1:MacArthur-Bates Communicative Development Inventory; DDT: Discrete Trial Teaching; EMT: Enhanced Milieu Teaching; IBR: Initiating Behavioral Requests; IJA: Initiating Joint Attention; JASP: JASPER: Joint Attention, Symbolic Play, Engagement and Regulation; MBCT: Melodic-Based Communication Therapy; MCT: Milieu Communication Training; NDWR: Number of Different Word RootsNLS: Naturalistic Language Sample; PLS-5: Preschool Language Scales Fifth Edition; RDLS: Reynell developmental language scales; RMIA: Rapid Motor Imitation Antecedent; SCU: Spontaneous Communicative Utterances; SGDs: Speech-Generating Devices; SIRS: Social Impression Rating Scale; SRT: Speech Repetition Therapy.

## 4. Discussion

In recent years, scientific attention to interventions aimed at MV autistic children has grown significantly. The results of this systematic review map the range of communication-based interventions available for MV autistic children and illustrate how these approaches are implemented across various developmental profiles and social contexts. Although the overall number of studies remains modest, the variety of intervention types, settings, and outcome domains offer valuable insight into how different strategies are applied to support language and social interaction in this population. A previous Cochrane review published in 2018 [4] included only two RCTs, reflecting the scarcity of high-quality evidence available at the time. Both studies were limited in sample size and scope, and no firm conclusions could be drawn regarding intervention efficacy. In contrast, the present review includes 18 studies. This substantial expansion in both quantity and methodological diversity indicates growing research attention and allows for a more comprehensive analysis of intervention types, outcomes, and implementation contexts. The current review thus provides a much-needed update to the field and helps orient future research directions. Unlike previous reviews, which primarily focused on randomized controlled trials or specific intervention models, the broader inclusion criteria adopted in the present review provide additional insights into the diversity of communication interventions currently available for minimally verbal autistic children. The inclusion of pilot, quasi-experimental, and observational studies allowed the identification of innovative and emerging approaches, as well as implementation strategies in real-world educational and clinical settings that would not have been captured by more restrictive reviews. While our findings are broadly consistent with the recent systematic review and meta-analysis by Pope et al. [16], which highlighted the effectiveness of NDBIs, the present review broadens these conclusions by showing that structured behavioral, AAC-based, music-based, and hybrid interventions may also provide potentially clinically meaningful benefits when tailored to children’s individual developmental profiles. Collectively, these findings support a personalized, multimodal approach rather than a single preferred intervention model.

Despite some variability across protocols, a consistent pattern emerged: many studies reported gains in expressive and receptive language skills, including word production, lexical diversity, and the emergence of early phrase structure [22,64]. These improvements were frequently accompanied by increases in spontaneous communicative behaviors such as verbal initiations, imitative attempts, and social reciprocity, underscoring the broader communicative impact of the interventions. Taken together, the evidence suggests that expressive vocabulary and spontaneous communication are the most consistently responsive domains, while play skills appear to be promising precursors and social-pragmatic abilities remain more resistant to change, often requiring more intensive or sustained intervention. However, these conclusions should be interpreted with caution, as the current evidence is based on a relatively small number of high-quality randomized controlled trials, generally modest sample sizes, and limited long-term follow-up. Moreover, the substantial clinical and methodological heterogeneity across studies precludes definitive conclusions regarding the superiority of any single intervention approach. Findings on phonological processing remain scarce but point to underexplored pathways that may hold future promise [65]. At the same time, variability in outcome measures continues to constrain comparability: standardized tests often fail to detect subtle progress, whereas NLS and caregiver reports are more sensitive to capturing functional change [6,12,66]. Several studies adopting NDBIs documented progress across multiple communicative domains, indicating that these approaches can foster both verbal language and social engagement. Interventions such as JASP+EMT and JASPER were delivered within contexts shaped by children’s spontaneous interests and participation, thereby targeting foundational abilities including joint attention, symbolic play, and natural language modeling. A consistent theme across these trials concerned the use of augmentative supports: when SGDs were introduced from the outset rather than delayed, children showed greater gains in spontaneous verbal output and social initiations. This pattern underscores the potential value of embedding assistive technologies early within socially meaningful exchanges, an observation that resonates with previous evidence suggesting that multimodal input can scaffold, rather than hinder, spoken language development [67]. These findings align with a broader body of research indicating that NDBIs promote language not through the direct teaching of isolated skills but by embedding communication in play-based, socially contingent contexts [68]. Moreover, adaptive designs further illustrate how tailoring interventions and incorporating multimodal supports can optimize spoken language trajectories. Parent-mediated approaches, such as FPI, add to this picture by showing that improvements in caregiver responsivity mediate children’s language outcomes, in line with longitudinal studies linking parental responsiveness to growth in joint engagement and vocabulary [69]. Collectively, these findings suggest that the effectiveness of NDBIs may derive from their ability to create developmentally rich, motivating contexts where communication and social interaction can emerge in tandem [70].

In contrast, more structured interventions such as DTT and RMIA showed targeted effectiveness, particularly for children with more limited receptive language or broader developmental delays. Although these programs may not elicit spontaneous language to the same extent as naturalistic models, they contribute essential groundwork by building basic communicative behaviors [71]. These include attention to linguistic stimuli, imitation, and responsiveness to prompts, which are often prerequisites for later development [72]. Overall, the results suggest that while emotional engagement and social context are crucial, they should be integrated with clear linguistic objectives to achieve comprehensive developmental outcomes [73]. The comparative results of NDBIs and structured approaches underscore the importance of aligning the intervention strategy with the child’s individual cognitive and communication profile, rather than adopting a one-size-fits-all model [72]. In addition to the variable methodological quality of the included studies, the substantial clinical and methodological heterogeneity across participant characteristics, intervention protocols, settings, and outcome measures further limits the ability to identify a single superior intervention approach. These findings therefore support the need for individualized, context-sensitive intervention planning rather than a universal treatment model. Several included studies further suggest that treatment responsiveness may be moderated by individual child characteristics, including cognitive functioning, baseline language abilities, joint attention skills, imitation ability, autism severity, and age. However, the inconsistent reporting of these variables across studies limits firm conclusions regarding their predictive value and highlights the need for more comprehensive participant characterization in future intervention research.

Expanding beyond established paradigms, innovative interventions incorporating rhythm, music, and motor imitation provide additional insight into how alternative pathways might support speech development in MV children. Protocols such as AMMT and MBCT, which combine rhythmic auditory stimuli with coordinated motor output, have demonstrated gains in phonemic accuracy, imitation, and novel word production [74,75]. These findings suggest that multi-sensory engagement can strengthen neural circuits associated with speech production and may facilitate lexical and phonological growth in MV learners [75]. Although the evidence remains preliminary, the approaches are theoretically grounded and resonate with embodied models of language development, which emphasize the role of motor engagement and sensory feedback in scaffolding early word learning [76]. Moreover, emerging results indicate that pre-treatment imitation skills may moderate responsiveness, highlighting the importance of individualized assignment when considering such interventions [72].

Few studies examined AAC. While these interventions did not consistently increase standardized scores of spoken language, they were associated with improvements in functional communication, reciprocity, and generalization across settings. Trials embedding PECS or tablet-based SGDs in classroom and peer-mediated contexts showed that children could achieve meaningful gains in communicative frequency and social engagement. Importantly, these findings suggest that intervention success should not be evaluated solely in terms of spoken language acquisition but also in terms of improvements in functional communication. The ability to express needs, preferences, and intentions in everyday contexts represents a clinically meaningful outcome that may substantially enhance participation and quality of life, even when spoken language gains are limited. These results reinforce the view that AAC should be conceptualized as a facilitator rather than an obstacle to speech development, particularly when implemented in socially interactive and ecologically valid environments [12].

Finally, few studies investigated hybrid or experimental models that incorporated orthographic supports, symbolic routines, or technology-mediated input. Although these approaches remain at an early stage of validation, they reflect a growing emphasis on personalized and scalable solutions. In particular, visually anchored or text-supported interventions may be especially promising for children with strong visual processing skills and limited oral output, with preliminary evidence suggesting benefits for vocabulary acquisition and communicative participation [77]. More broadly, these models exemplify the field’s shift toward flexible frameworks that can be adapted to the diverse cognitive and communicative profiles of MV autistic children [6].

Most RCTs were rated “fair” (33.3%) or “poor” (46.7%) quality, primarily due to unclear allocation concealment and high selective reporting risk (40.0% of studies). The three “good quality” RCTs [54,59,60] all reported improvements in expressive language and spontaneous communication, supporting confidence in these findings. Outcomes related to play skills and social communication were more variable and derived predominantly from lower-quality studies and should be interpreted cautiously. The non-randomized studies (two “poor”, one “fair”) are considered supplementary and do not alter the primary conclusions. In summary, this review provides converging evidence that early, socially embedded, and multimodal interventions can produce measurable gains in expressive language and related communication skills in MV autistic children. This aligns with broader developmental literature showing that language and social communication emerge through the interplay of environmental supports, cognitive capacities, and individual learning pathways [78]. Overall, the most consistent evidence currently suggests improvements in expressive language and spontaneous communicative behaviors. These outcomes were consistently reported across multiple studies and were supported by the higher-quality randomized controlled trials included in this review.

Regarding treatment modalities, NDBIs appear to have the strongest evidence base among the intervention approaches identified in this review. These approaches utilize child-led, play-based contexts rich in authentic social interactions, fostering functional and generalizable communicative skills. Early introduction of augmentative communication devices, active caregiver involvement, and structured interventions provide complementary benefits but typically show more targeted and less broadly generalized efficacy. Nevertheless, these conclusions should be interpreted with caution given the limited number of high-quality randomized controlled trials, generally modest sample sizes, limited long-term follow-up, and the substantial heterogeneity across studies, which precludes definitive conclusions regarding the superiority of any single intervention approach. Taken together, these findings emphasize the importance of developing personalized, early, and multimodal models of care tailored to the individual developmental profiles and real-life contexts of MV autistic children to maximize communicative growth. Strengthening methodological rigor while advancing such personalized, context-sensitive interventions will be critical to ensuring scalable and effective models of care in this population.

## 5. Limitations

This systematic review highlights several key limitations that must be considered when interpreting the findings. One major issue is the inconsistent and ambiguous definition of “minimally verbal” across studies. As noted by Tager-Flusberg et al. [66], the lack of a shared operational definition limits the accumulation of evidence. Although a threshold of ≤20 words was most commonly used, variability across studies (e.g., <10 to <50 words) and inconsistent qualitative criteria (such as “functional” or “intelligible” speech) reduce comparability. Another limitation concerns the search terms adopted for the literature search. Although the search strategy was developed according to the registered PROSPERO protocol and supplemented by manual screening of the reference lists of included studies and relevant reviews, terminology describing minimally verbal autistic individuals has evolved over time. Earlier studies may have described this population using alternative terms such as “nonverbal autism”, “limited speech”, “low-verbal”, or “preverbal”. Therefore, the adopted search terminology may represent a potential limitation of the present review, as some earlier studies may have used different descriptors for this population. A further limitation concerns the potential for publication bias. Because a formal assessment of publication bias was not feasible due to the substantial heterogeneity of study designs, interventions, and outcome measures, it is possible that studies reporting null or negative findings were underrepresented. Consequently, the overall effectiveness of the interventions included in this review may have been overestimated. In addition, the clinical characterization of samples was often insufficient. Information on NVIQ and autism severity was frequently underreported, making it difficult to evaluate differential treatment effects across neurodevelopmental profiles. This limited phenotypic characterization also reduces sensitivity to variability within ASD, including potential gender differences. Males were overrepresented, and no studies have directly compared treatment effectiveness between sexes. From a methodological perspective, the heterogeneity of interventions, outcome measures, and contexts prevented meta-analysis, leading to a primarily narrative synthesis. Another important limitation concerns the geographic concentration of studies, with most conducted in the United States. This raises concerns about the generalizability of findings to other cultural, educational, and healthcare contexts, highlighting the need for more cross-cultural and international research.

## 6. Practical and Future Implications

This review emphasizes the importance of adopting individualized and flexible intervention strategies tailored to the specific communicative profiles of MV autistic children. Multimodal approaches that combine naturalistic teaching methods, augmentative communication technologies, and active caregiver involvement appear particularly promising [79]. However, several challenges remain for large-scale implementation. It is crucial to establish standardized diagnostic and functional criteria to consistently identify MV children across research and clinical contexts, enabling better comparison of findings and service provision. Additionally, more longitudinal research is needed to evaluate the generalization and long-term impact of interventions as children develop and face more complex social demands. Furthermore, comprehensive characterization of study samples is essential to distinguish subgroups within the MV population [8].

From an implementation perspective, future interventions should prioritize accessibility, sustainability, and adaptability to everyday contexts such as homes and schools. This is especially important in low-resource settings, where community-based models and scalable technologies can improve access to care [80]. Finally, intervention outcome measures should extend beyond language production to include broader aspects of development, such as social interaction, emotional regulation, and overall quality of life, providing a more comprehensive evaluation of effectiveness.

## Data Availability

No new data were created or analyzed in this study. Data sharing is not applicable to this article.

## Supplementary Materials

The following supporting information can be downloaded at: https://www.mdpi.com/article/10.3390/medsci14030388/s1, Table S1: PRISMA 2020 statement and checklist; Supplement S1: Literature search; Method S1: Eligibility Criteria and Data extraction and evaluation; Table S2: Literature References; Table S3: Sample characteristics and assessment measures for MV autistic children; Table S4: Overview of intervention characteristics; Table S5: Domain-by-domain RoB assessment for the included randomized controlled trials, with corresponding AHRQ overall study quality; Table S6: ROBINS-I risk-of-bias assessment for included non-randomized studies, with AHRQ overall study quality.

## Abbreviations

The following abbreviations are used in this manuscript:

AAC	Augmentative and alternative communication
ASD	Autism Spectrum Disorder
MV	Minimally verbal
NDBI	Naturalistic Developmental Behavioral Intervention
RCT	Randomized controlled trials
NVIQ	Nonverbal Intellectual Quotient
ARHQ	Agency for Healthcare Research and Quality
EMT	Enhanced Milieu Teaching
SGDs	Speech-Generating Devices
JASPER	Joint Attention, Symbolic Play, Engagement and Regulation
FPI	Focused Playtime Intervention
PRT	Pivotal Response Treatment
MCT	Milieu Communication Training
RMIA	Rapid Motor Imitation Antecedent
AMMT	Auditory-Motor Mapping Training
MBCT	Melodic-Based Communication Therapy
PECS	Picture Exchange Communication System
