# Supplementary material for: Interventions to Promote Communication in Minimally Verbal Autistic Children: A Systematic Review
Source: Med Sci (Basel). 2026 Jul 13;14(3):388. doi: 10.3390/medsci14030388 (PMC13414371; doi:10.3390/medsci14030388)
Supplement: Supplementary file 1 [file medsci-14-00388-s001.zip › medsci-4374413-supplementary.pdf]

SUPPLEMENTARY MATERIAL

Table S1: PRISMA 2020 statement and checklist …..…..…..…..…..…..…..…..…..…..……...…..…..……..…..…..……………..page 2-4

Supplement S1: Literature search..…..…..…....…..…...…....…..…...………...…...…..…...…...…..…...…....…..…...……………page 5-6

Method S1: Eligibility Criteria and Data extraction and evaluation………………………………………………………………. page 7-8

Table S2: Literature References………………………………………………………………………………………………… page 9-10

Table S3: Sample characteristics and assessment measures for MV autistic children………………………………………………. page 11-17

Table S4: Overview of intervention characteristics……………………………………………………………………………. page 18-25

Table S5: Domain-by-domain RoB assessment for the included randomised controlled trials, with corresponding AHRQ overall study quality…… page 26

Table S6: ROBINS-I risk-of-bias assessment for included non-randomised studies, with AHRQ overall study quality…………………… page 27

Table S1: PRISMA 2020 statement and checklist ***(definite pending)***

Section and topic	Item #	Checklist item	Location where item is reported
**TITLE**			
Title	1	Identify the report as a systematic review or a meta-analysis	Title
**ABSTRACT**			
Structured summary	2	See the PRISMA 2020 for Abstracts checklist (table 2)	Abstract
**INTRODUCTION**			
Rationale	3	Describe the rationale for the review in the context of existing knowledge.	Introduction
Objectives	4	Provide an explicit statement of the objective(s) or question(s) the review addresses.	Introduction
**METHODS**			
Eligibility criteria	5	Specify the inclusion and exclusion criteria for the review and how studies were grouped for the syntheses.	Methods
Information sources	6	Specify all databases, registers, websites, organisations, reference lists and other sources searched or consulted to identify studies. Specify the date when each source was last searched or consulted.	Methods
Search strategy	7	Present the full search strategies for all databases, registers and websites, including any filters and limits used.	Methods, Supplement 1
Selection process	8	Specify the methods used to decide whether a study met the inclusion criteria of the review, including how many reviewers screened each record and each report retrieved, whether they worked independently, and if applicable, details of automation tools used in the process.	Methods
Data collection process	9	Specify the methods used to collect data from reports, including how many reviewers collected data from each report, whether they worked independently, any processes for obtaining or confirming data from study investigators, and if applicable, details of automation tools used in the process.	Methods, supplement_figure S1
Data items	10a	List and define all outcomes for which data were sought. Specify whether all results that were compatible with each outcome domain in each study were sought (e.g. for all measures, time points, analyses), and if not, the methods used to decide which results to collect.	Methods

	10b	List and define all other variables for which data were sought (e.g. participant and intervention characteristics, funding source). Describe any assumptions made about any missing or unclear information.	Methods
Study risk of bias assessment	11	Specify the methods used to assess risk of bias in the included studies, including details of the tool(s) used, how many reviewers assessed each study and whether they worked independently, and if applicable, details of automation tools used in the process.	Methods
Effect measures	12	Specify for each outcome the effect measure(s) (e.g. risk ratio, mean difference) used in the synthesis or presentation of results.	Methods
Synthesis methods	13a	Describe the processes used to decide which studies were eligible for each synthesis (e.g. tabulating the study intervention characteristics and comparing against the planned groups for each synthesis (item #5)).	Methods
	13b	Describe any methods required to prepare the data for presentation or synthesis, such as handling of missing summary statistics, or data conversions.	Methods
	13c	Describe any methods used to tabulate or visually display results of individual studies and syntheses.	Methods
	13d	Describe any methods used to synthesise results and provide a rationale for the choice(s). If meta-analysis was performed, describe the model(s), method(s) to identify the presence and extent of statistical heterogeneity, and software package(s) used.	Methods
	13e	Describe any methods used to explore possible causes of heterogeneity among study results (e.g. subgroup analysis, metaregression).	Methods
	13f	Describe any sensitivity analyses conducted to assess robustness of the synthesised results.<br>Reporting bias assessment.	Methods
Reporting bias assessment	14	Describe any methods used to assess risk of bias due to missing results in a synthesis (arising from reporting biases).	Methods
Certainty assessment	15	Describe any methods used to assess certainty (or confidence) in the body of evidence for an outcome	Does not apply
**RESULTS**			
Study selection	16a	Describe the results of the search and selection process, from the number of records identified in the search to the number of studies included in the review, ideally using a flow diagram (see fig 1).	Results, Figure S1
	16b	Cite studies that might appear to meet the inclusion criteria, but which were excluded, and explain why they were excluded.	Results, Figure S1
Study characteristics	17	Cite each included study and present its characteristics.	Results, Table S2; Table 1, 2
Risk of bias within studies	18	Present assessments of risk of bias for each included study	Result, Table 3

Results of individual studies	19	For all outcomes, present, for each study: (a) summary statistics for each group (where appropriate) and (b) an effect estimate and its precision (e.g. confidence/credible interval), ideally using structured tables or plots.	Does not apply
Results of syntheses	20a	For each synthesis, briefly summarise the characteristics and risk of bias among contributing studies.	Result, Table S1, Table 1, 2, 3
	20b	Present results of all statistical syntheses conducted. If meta-analysis was done, present for each the summary estimate and its precision (e.g. confidence/credible interval) and measures of statistical heterogeneity. If comparing groups, describe the direction of the effect.	Does not apply
	20c	Present results of all investigations of possible causes of heterogeneity among study results.	Results, Table 2
	20d	Present results of all sensitivity analyses conducted to assess the robustness of the synthesised results	Results, Table 2
Reporting biases	21	Present assessments of risk of bias due to missing results (arising from reporting biases) for each synthesis assessed.	Result Table 3
Certainty of evidence	22	Present assessments of certainty (or confidence) in the body of evidence for each outcome assessed.	Results
**DISCUSSION**			
Discussion	23a	Provide a general interpretation of the results in the context of other evidence.	Discussion
	23b	Discuss any limitations of the evidence included in the review.	Discussion
	23c	Discuss any limitations of the review processes used.	Discussion
	23d	Discuss implications of the results for practice, policy, and future research.	Discussion
**OTHER INFORMATION**			
Registration and protocol	24a	Provide registration information for the review, including register name and registration number, or state that the review was not registered.	Methods
	24b	Indicate where the review protocol can be accessed, or state that a protocol was not prepared.	Methods
	24c	Describe and explain any amendments to information provided at registration or in the protocol.	Does not apply
Support	25	Describe sources of financial or non-financial support for the review, and the role of the funders or sponsors in the review.	Funding
Competing interests	26	Declare any competing interests of review authors.	Conflict of interests
Availability of data, code, and other materials	27	Report which of the following are publicly available and where they can be found: template data collection forms; data extracted from included studies; data used for all analyses; analytic code; any other materials used in the review.	Supplementary File 1

Supplement S1: Literature search

Two seasoned information specialists (RP and AP) crafted the search strategy in accordance with the PRISMA guideline for reporting. Keywords were carefully curated based on expert opinions. The following electronic databases and international trial registries were searched Embase, Web of Science, Cochrane Library, and PubMed (National Library of Medicine). Search syntax for each database (in alphabetical order)

A. EMBASE

The search strategy was defined by identifying four mesh terms related to: ('autism'/exp OR autism) AND ('minimally verbal' OR (minimally AND verbal)) and ('autism'/exp OR autism) AND ('minimally speaking' OR (minimally AND ('speaking'/exp OR speaking))).

B. COCHRANE LIBRARY

The search strategy was defined by identifying four mash terms related to: *((ALL=(autism)) AND ALL=(minimally verbal)) and ((ALL=(autism)) AND ALL=(minimally speaking))*

C. PUBMED

The search strategy was defined by identifying four mash terms related to: ("autism s"[All Fields] OR "autisms"[All Fields] OR "autistic disorder"[MeSH Terms] OR ("autistic"[All Fields] AND "disorder"[All Fields]) OR "autistic disorder"[All Fields] OR "autism"[All Fields]) AND "minimally"[All Fields] AND ("verbal"[All Fields] OR "verbalization"[All Fields] OR "verbalizations"[All Fields] OR "verbalize"[All Fields] OR "verbalized"[All Fields] OR "verbalizer"[All Fields] OR "verbalizers"[All Fields] OR "verbalizing"[All Fields] OR "verbally"[All Fields]) and ("autism s"[All Fields] OR "autisms"[All Fields] OR "autistic disorder"[MeSH Terms] OR ("autistic"[All Fields] AND

"disorder"[All Fields]) OR "autistic disorder"[All Fields] OR "autism"[All Fields]) AND "minimally"[All Fields] AND ("speak"[All Fields] OR "speaking"[All Fields] OR "speaks"[All Fields])

D. WEB OF SCIENCE

The search strategy was defined by identifying four mash terms related to: *((ALL=(autism)) AND ALL= (minimally verbal)) and ((ALL=(autism)) AND ALL= (minimally speaking))*

Methods S1

Eligibility Criteria

Studies were included if they: (a) were published in peer-reviewed journals; (b) used RCT (parallel or crossover), non-randomized, pilot, or observational designs evaluating communication-based interventions; (c) involved participants with a formal ASD diagnosis according to DSM-5 (*Diagnostic and Statistical Manual of Mental Disorders: DSM-5^{TM}, 5th Ed*, 2013), DSM-IV (American Psychiatric Association, 2000), or the International Classification of Diseases (ICD-9/ICD-10) (Slee, 1978; World Health Organization, 2004); (d) included minimally verbal (MV) individuals under 18 years; and (e) were published in English or Italian, or had an available English translation.

Studies were excluded if they: (a) were case reports, letters, editorials, conference abstracts, reviews, or book chapters; (b) did not clearly define ASD diagnosis; (c) included non-MV participants or individuals aged ≥18 years; (d) reported overlapping samples (the most complete dataset was retained); or (e) were published in other languages without English translation.

Data extraction and evaluation

To describe and analyze study characteristics, all variables were coded as categorical and defined by one author, with intermediate and final checks to ensure consistency. Two authors independently reviewed the extracted data for adherence to the coding framework. Discrepancies were resolved by consensus, and final agreement was reached for all included studies.

Data extraction covered:

a) General study information (first author, year, country);
b) Sample characteristics (size, gender, age, diagnostic procedures, nonverbal intellectual quotient [NVIQ], ASD severity, and co-occurring conditions, when available);
c) Intervention characteristics (type, frequency, duration, setting, outcome measures, and treatment goals).

Reference

American Psychiatric Association (2000) *Diagnostic and Statistical Manual of Mental Disorders, Fourth Edition, Text Revision (DSM-IV-TR)*. 4th edn Arlington, VA: American Psychiatric Association. Available at: http://www.psychiatryonline.com/resourceTOC.aspx?resourceID=1 (accessed 9 April 2026).

Diagnostic and Statistical Manual of Mental Disorders: DSM-5^{TM}, 5th Ed (2013) Diagnostic and statistical manual of mental disorders: DSM-5^{TM}, 5th ed. Arlington, VA, US: American Psychiatric Publishing, Inc.

Slee VN (1978) The International Classification of Diseases: ninth revision (ICD-9). *Annals of Internal Medicine* 88(3): 424–426.

World Health Organization (ed.) (2004) *International Statistical Classification of Diseases and Related Health Problems*. 10th revision, 2nd edition. Geneva: World Health Organization.

Table S2. Literature References

ID	Authors	Year	Title	Journal	Continent - Country	Study Design
1	Abdi S. et al.	2023	The Effect of Combined Intervention on Improvement of Early Lexical Development in Minimally Verbal Children with Autism Spectrum Disorder.	Medical journal of the Islamic Republic of Iran	**Asia** – Iran	Within-subject
2	Almirall D. et al.	2016	Longitudinal Effects of Adaptive Interventions With a Speech-Generating Device in Minimally Verbal Children With ASD	Journal of clinical child and adolescent psychology	**USA** – California – Maryland - Michigan - Tennessee – Utah	RCT – SMART design
3	Barrett, AC et al.	2020	Social responsiveness and language use associated with an enhanced PRT approach for young children with ASD: results from a pilot RCT of the PRISM model	Research in autism spectrum disorders	**USA** – California - South Carolina	RCT (pilot)

4	Bauminger-Zviely, N. et al.	2020	Communicating Without Words: school-Based RCT Social Intervention in Minimally Verbal Peer Dyads with ASD	Journal of clinical child and adolescent psychology	**Asia** – Israele	RCT
5	Chang YC et al.	2018	Symbolic Play in School-Aged Minimally Verbal Children with Autism Spectrum Disorder	Journal of autism and developmental disorders	**USA** – California – Maryland - Tennessee	RCT
6	Chenausky, K.V. et al.	2022	Auditory-motor mapping training: Testing an intonation-based spoken language treatment for minimally verbal children with autism spectrum disorder	Ann. New York Acad. Sci.	**USA** – Massachusetts	RCT
7	Clark, G. T. et al.	2023	Word Learning With Orthographic Support in Nonspeaking and Minimally Speaking School-Age Autistic Children.	Journal of speech, language, and hearing research	**USA** - New York<br>**Europe** – Sweden	Within-subject
8	Goods KS et al.	2013	Preschool based JASPER intervention in minimally verbal children with autism: pilot RCT.	Journal of autism and developmental disorders	**USA** – California	RCT (pilot)
9	Hampton, L.H. et al.	2020	Multi-component communication intervention for children with autism: A randomized controlled trial	Autism	**USA** – Tennessee – Texas	RCT
10	Howlin et al.	2007	The effectiveness of Picture Exchange Communication System (PECS) training for teachers of children with autism: a pragmatic, group randomised controlled trial	Journal of Child Psychology and Psychiatry	**Europe**-United Kingdom	RCT
11	Kasari C. et al	2014	Communication interventions for minimally verbal children with autism: a sequential multiple assignment randomized trial	Journal of the American Academy of Child and Adolescent Psychiatry	**USA** – California – Maryland -<br>Michigan - Tennessee – Utah	RCT – SMART design
12	Kasari, C. et al.	2023	Spoken language outcomes in limited language preschoolers with autism and global developmental delay: RCT of early intervention approaches.	Autism research	**USA** - California - Maryland - New York - Oregon	RCT
13	Paul R. et al.	2013	Comparing spoken language treatments for minimally verbal preschoolers with autism spectrum disorders.	Journal of autism and developmental disorders	**USA** – Connecticut - New York<br>**Europe** – Greece	Quasi-experimental
14	Sandiford, GA et al.	2013	A Pilot Study on the Efficacy of Melodic Based Communication<br>Therapy for Eliciting Speech in Nonverbal Children with Autism	Journal of autism and developmental disorders	**USA**-California	RCT (pilot)
15	Schreibman, LE et al.	2014	A Randomized Trial Comparison of the Effects of Verbal and	Journal of autism and developmental disorders	**USA**- California	RCT

			Pictorial Naturalistic Communication Strategies on Spoken<br>Language for Young Children with Autism			
16	Siller et al.	2013	A parent-mediated intervention to increase responsive parental behaviors and child communication in children with ASD: a randomized clinical trial	Journal of Autism and Developmental Disorders	**USA** - California	RCT
17	Thiemann-Bourque K. et al.	2018	Incorporating a Peer-Mediated Approach Into Speech-Generating Device Intervention: Effects on Communication of Preschoolers With Autism Spectrum Disorder	Journal of speech, language, and hearing research	**USA** – Kansas	RCT
18	Yoder PJ et al.	1988	Speech following sign language training in autistic children with minimal verbal language.	Journal of autism and developmental disorders	**USA** – Tennessee	RCT

Table S3. Sample characteristics and assessment measures for MV autistic children

ID	Sample Size [M:F]	Ethnicity	M:F	Age Mean (SD);[Range]	Severity of Autism	ADOS	Comorbidity	Non-verbal I.Q. Mean (SD)	Definition of MV	Measures MV
1	10 [7M:3F]	N.S.	2.333:1	47.9 Months (8.3); [3-6 years]	Mild: 3 Moderate : 5 Severe: 2	N.S.	N.S.	N.S.	Less than 50 functional words	CDI-I
2	61 [51M:10 F]*	52% Non White(Caucasian vs. Other)	5.1:1	6.31 Years (1.16); [5-8 Years]	N.S.	20.07 (SD 4.4)	N.S.	68.2 (19.0)	Fewer than 20 spontaneous new words	NLS: transcription and linguistic coding of utterances and spontaneous words
3	21[18M: 3F] (12 treatment, 9 control)	67% White, 14% Hispanic/Latino, 5% Black, 14% Multiracial /Other	6:1	3.87 Years (0.71); [1.5–4.5 Years]	N.S.	N.S.	N.S.	81.95 (17.73)	<30 function words in initial parent-infant play, according to Kasari et al. (2013) criteria; speech limited to 1–2 functions, often echolalic verbalization	Parent-child interaction video coding: • NTW • NDW • MLU • Social responsiveness
4	54 [44M:10 F] (18 in *conversation*, 18 in *collaboration*, 18 in *waitlist control*)	N.S.	4.4:1	Conversation = 122.61 (18.61) Collaboration = 134.76 (28.56) Control = 136.17 (22.52) [8–16 Years]	N.S.	N.S.	Intellectual disability	Conversation: 62.28 (20.65), Collaboration: 71.22 (24.97), Control: 54.17 (13.15)	<30 function words in initial parent-infant play, according to Kasari et al. (2013) criteria;	Communication was assessed through multiple methods: (1) SCS-A for dyadic interaction quality, (2) PPTV-III teacher reports of spontaneous word use, (3) for receptive language, and (4) structured video observations coding gestures, vocalizations, and use of augmentative tools.
5	58**	JASP+ EMT: African	N.S.	JASP+EMT	N.S.	JASP+EMT	N.S.	JASP + EMT	< 20 different spontaneous words in 20 minutes (NLS)	NLS

*Almirall et al. (2016) used a SMART design. All participants received initial PRT for 12 weeks; non-responders were randomized to continued PRT or PRT + SGD for the second phase.
**Participants were randomized to two groups (JASP+EMT and JASP+EMT+AAC), but exact numbers per group were not reported.
*** SA: Social Afffect

		American: 21%<br>Caucasian: 47%<br>Hispanic: 4%<br>Asian: 21%<br>Other: 7%<br><br>JASP+EMT+AAC:<br>African American: 26%<br>Caucasian: 48%<br>Hispanic: 7%<br>Asian: 16%<br>Other: 3%		6.45 Years (1.11); [5–8 years]<br><br>JASP+EMT+AAC:<br>6.65 Years (1.23); [5–8 years]		15.61 (SD = 3.91)<br><br>JASP + EMT + AAC15.50 (SD = 3.86)		4.01 (1.10) - mental age in years<br><br>JASP + EMT + AAC<br>4.07 (1.14) - mental age in years		
6	14 (AMMT 6M:2F; SRT 5M:1F)	N.S.	AMMT 3:1<br>SRT 5:1	AMMT<br>7.0 Years (1.11) [5-11 Years]<br>SRT<br>6.5 Years (1.0) [5-11 Years]	N.S.	AMMT<br>ADOS 22.8<br>SRT<br>ADOS 23.3	N.S.	AMMT<br>68.0 (8.9)<br>SRT<br>70.5 (8.6)	Expressive vocabulary ≤ 20 words	ADOS-2 Module 1; expressive vocabulary count during ADOS
7	22 [18M:4F]	12 White, 8 Asian, 2 Hispanic/Latino	4.5:1	11.99 Years (3.57); [6.2 – 17.2 Years]	N.S.	N.S.	N.S.	N.S.	Fewer than 30 spontaneously spoken words	Parent report of daily spoken word count, collected via Background Questionnaire; classification based on Koegel et al. (2020).

*Almirall et al. (2016) used a SMART design. All participants received initial PRT for 12 weeks; non-responders were randomized to continued PRT or PRT + SGD for the second phase.
**Participants were randomized to two groups (JASP+EMT and JASP+EMT+AAC), but exact numbers per group were not reported.
*** SA: Social Afffect

8	6 [N.S] Control<br>5 [N.S] Treatment	over half of the children identified as African-American, Hispanic, or Asian	N.S.	51.9 months (N.S) [3-5 Years]	N.S.	N.S.	N.S.	15.45 months – Mental Age in months	less than ten spontaneous, functional and communicative words	Parent and teacher report, verified at baseline assessment
9	34 Control [27M:7F]<br>34 Treatment [26M:8F]	52% White Control<br><br>64% White Treatment	3.8:1 Control<br>3.25:1 Treatment	Control<br>43 Months (6)<br>[36–55 Months]<br>Treatment<br>43 Months (5)<br>[36–57 Months]	CSS Control 7.4 (1.7; 5–10)<br>Treatment 8.0 (1.7; 4–10)	ADOS Control 30 (SD=5)<br>ADOS Treatment 21(SD=5)	N.S.	N.S.	fewer than 20 different spontaneous words during a 20-min language sample	NLS, coded with SALT software.
10	ITG: 26 [21M:5F]<br><br>DTG: 30 [27M:3F]<br><br>NTG:28 [25M:3F]	N.S.	ITG: 4.2:1<br><br>DTG: 9:1<br><br>NTG: 8.3:1	ITG: 73.1 months (15.8) [47.3–106.3]<br>DTG: 86.6 months (12.7) [62.0–113.5]<br><br>NTG: 85.6 months (13.6) [61.0–122.1]	N.S.	ITG: 16.4 (2.7)<br><br>DTG: 16.9 (2.9)<br><br>NTG: 15.3 (3.2)	N.S.	ITG: 25.9 (11.4)<br><br>DTG: 22.7 (8.2)<br><br>NTG: 27.3 (10.2)	single words or word approximations	ADOS-G<br>MSLE

*Almirall et al. (2016) used a SMART design. All participants received initial PRT for 12 weeks; non-responders were randomized to continued PRT or PRT + SGD for the second phase.

**Participants were randomized to two groups (JASP+EMT and JASP+EMT+AAC), but exact numbers per group were not reported.

*** SA: Social Afffect

11	30 JASP+EMT [26M:4F]<br>31 JASP + EMT + SGD [25M:6F]	JASP + EMT<br>White: 14<br>African-American: 6<br>Asian American: 7<br>Hispanic: 1<br>Other: 2<br><br>JASP+EMT+SGD<br>White: 15<br>African-American: 5<br>Asian American: 5<br>Hispanic: 2<br>Other: 1	JASP + EMT<br>6.5:1<br><br>JASP+ EMT+ SGD<br>5.2:1	JASP+EMT<br>6.18 Years (1.08)<br>[5 - 8 years]<br><br>JASP+EMT+SGD<br>6.44 Years (1.23) [5 - 8 years]	N.S.	Overall: 20.02 (SD 4.37);<br>JASP+EMT: 20.60 (SD 4.47);<br>JASP+EMT+SGD: 19.55 (SD 4.27)	N.S.	JASP + EMT<br>68.73 (N.S.)<br><br>JASP+EMT+SGD<br>67.65 (N.S.)	fewer than 20 spontaneous different words used during the 20-minute NLS	NLS
12	DTT 82 [70M:12F]<br><br>JASPER 82 [67M:15F]	DDT<br>Hispanic:20<br>Non Hispanic:62<br><br>JASPER<br>Hispanic: 13<br><br>Non Hispanic: 69	DTT<br>5.8:1<br><br>JASPER<br>4.6:1	DTT 44.33 (5.45) [33-54 months]<br><br>JASPER 45.09 (5.23) [33-54 months]	DTT CSS: 7.32 (SD = 1.61)<br><br>JASPER CSS: 7.40 (SD = 1.65)	N.S.	N.S.	DTT 56.44 (12.32)<br><br>JASPER 55.36 (14.58)	fewer than 30 spontaneous words across entry assessments	ADOS-2<br>MSEL<br>RDLS

*Almirall et al. (2016) used a SMART design. All participants received initial PRT for 12 weeks; non-responders were randomized to continued
PRT or PRT + SGD for the second phase.
**Participants were randomized to two groups (JASP+EMT and JASP+EMT+AAC), but exact numbers per group were not reported.
*** SA: Social Afffect

13	10 RMIA [6M:4F] 12 MCT [11M:1F]	N.S.	RMIA 1.5:1<br><br>MCT 11:1	RMIA 4.3 Years (1.2) [N.S]<br><br>MCT 3.5 Years (0.8) [N.S]	N.S.	RMIA: 14.0 ADOS-SA***<br><br>MCT: 14.6 ADOS-SA	N.S.	RMIA 22.6 (4.6) Months<br><br>MCT 22.2 (5.6) Months	spontaneous expressive vocabulary by parent report of fewer than 15 words	CSBS-Caregiver Questionnaire; CSBS-Behavioral Observation; VABS-II Expressive Language Subscale
14	5 [5M:0F] Tradiotional<br><br>7 [6M:1F] MBCT	N.S.	Tradiotional 5:0<br><br>MBCT 6:1	Traditional 5.8 years (.8) [5-7 years]<br><br>MCBT 5.9 (.9) [5-7 years]	N.S.	Traditional ADOS total score 17<br><br>MCBT ADOS total score 20	N.S.	N.S.	expressive vocabulary of no more than 10 words which were not used on a daily basis and having no functional speech.	Parent Survey
15	39 (PRT = 20 [18M:2F]; PECS = 19 [16M:3F])	N.S.	PRT 9:1<br><br>PECS 5.33:1	PRT: 29.5 Months (6.9) [20-45 months]<br><br>PECS: 28.9 Months (4.2) [20-45 months]	N.S.	N.S.	N.S.	Cognitive level categorized as "low" or "high" using MSEL Visual Reception subscale; no mean/SD IQ reported	no more than nine intelligible words	VABS, MSEL, ADOS, EOWPVT, 25-min parent–child observation
16	70 (36 intervention, 34 control) [64M:6F]	Experimental Group: Hispanic/Latino:17 White:8 Asian:4 Black:3 Mixed:4<br><br>Control Group:	10.6:1	Experimental Group: 58.3 months (12.7) [33-82]<br><br>Control Group: 55.9 months (11.9) [32-76]	N.S.	Experimental Group: 19.6<br><br>Control Group: 20.6	Cerebral Palsy (n=2); history of seizures (n=10)	N.S.	Fewerthan 25 words and no phrases	N.S.

*Almirall et al. (2016) used a SMART design. All participants received initial PRT for 12 weeks; non-responders were randomized to continued PRT or PRT + SGD for the second phase.

**Participants were randomized to two groups (JASP+EMT and JASP+EMT+AAC), but exact numbers per group were not reported.

*** SA: Social Afffect

		Hispanic/Latino:14<br>White:6<br>Asian:9<br>Black:2<br>Mixed:3								
17	45 (Treatment 23 [16M:7F]; Control 22[20M:2F]	Treatment: White 12, African American 8, other/mixed 3;<br>Control: White 13, African American 7, Hispanic 1, other/mixed 1	Treatment 2.28:1<br><br>Control 10:2	Treatment 48 months (N.S) [37-60]<br><br>Control 46 months (N.S) [35-58]	N.S.	N.S.	N.S.	MSEL Early Learning Composite (standard score): **Treatment** = 49.1; **Control** = 50.0 (N.S) [49–63]	Less than 20 spontaneous words	N.S.
18	Speech Alone 15<br><br>Sign Alone 15<br><br>Simultaneous Sign and Speech 15<br><br>Alternating Sign and	N.S.	N.S.	Speech Alone 5.4 years (1.7) [N.S]<br><br>Sign Alone 5.6 years (2.0) [N.S]<br><br>Simultaneous Sign and Speech 5.0 years (1.2) [N.S]<br><br>Alternating Sign and Speech 5.4 years (2.1) [N.S]	CARS: moderate –severe	N.S.	N.S.	Speech Alone 42.9 (17.8)<br><br>Sign Alone 40.5 (33.1)<br><br>Simultaneous Sign and Speech 41.0 (23.8)<br><br>Alternating Sign and Speech 44.4 (24.4)	expressive vocabulary of 25 words or less	Parent Questionnaire

*Almirall et al. (2016) used a SMART design. All participants received initial PRT for 12 weeks; non-responders were randomized to continued PRT or PRT + SGD for the second phase.
**Participants were randomized to two groups (JASP+EMT and JASP+EMT+AAC), but exact numbers per group were not reported.
*** SA: Social Afffect

	Speech 15									

AAC: Augmentative or Alternative communication; ADOS: Autism Diagnostic Observation Schedule; ADOS-G: Autism Diagnostic Observation Schedule-Generic; AMMT: Auditory-Motor Mapping Training; CARS: Childhood Autism Rating Scale; CDI-I: MacArthur-Bates Communicative Development Inventory; CSS: Calibrated severity score; DTG: Delayed Treatment Group; EMT: Enhanced Milieu Teaching; EOWPVT: Expressive One-Word Picture Vocabulary Test; F: Female; ITG: Immediate Treatment Group; JASP: JASPER: Joint Attention, Symbolic Play, Engagement and Regulation; M: Male; MBCT: Melodic-Based Communication Therapy; MCT: Milieu Communication Training; MLU: Mean Length of Utterance; MSLE: Mullen Scales of Early Learning; N.S.: Not specificated; NDW: Number of Different Words; NLS: Naturalistic Language Sample; NTG: No Treatment Group; NTW: Total Number of Words; PECS: Picture Exchange Communication System; : PPVT: Peabody Picture Vocabulary Test–III; PRT: Pivotal Response Treatment; RMIA: Rapid Motor Imitation Antecedent; SCS-A: Self-compassion Scale – A; SGDs: Speech-Generating Devices; SRT: Speech Repetition Therapy; VABS: Vineland Adaptive Behavior Scales

*Almirall et al. (2016) used a SMART design. All participants received initial PRT for 12 weeks; non-responders were randomized to continued PRT or PRT + SGD for the second phase.
**Participants were randomized to two groups (JASP+EMT and JASP+EMT+AAC), but exact numbers per group were not reported.
*** SA: Social Afffect

Table S4. Overview of intervention characteristics

ID	Aims	Type of Intervention	Setting	Intervention Provider	Total N. of Experimental Sessions	Duration in Weeks (Frequency of Sessions/Week)	Session Time (Min)
**1**	Study on a combined intervention (behaviorism, schema theory, sociocultural theory, event representation) to improve vocabulary in autistic MV	The combined intervention included different methods based on contemporary behaviorism, schema theory, sociocultural theory, and event representation approaches.	Speech therapy clinics, Iran University of Medical Sciences	Clinician + Parent mediated (mixed)	16	8 Weeks<br>(2 sessions/week)	45
**2**	Compare communication growth among three adaptive interventions using JASP+EMT with/without SGDs in autistic MV children.	Adaptive interventions: JASP+EMT and JASP+EMT+SGD	University-affiliated clinical centers (UCLA, Vanderbilt, Kennedy Krieger)	Clinician deliverd + parent implemented (mixed)	48 (early responders) or 60 (slow responders)	24 weeks (2–3 sessions/week depending on treatment response<br>•Phase 1: 2s/w<br>•Phase 2: 2 or 3s/w)	60

**3**	Evaluate impact of PRISM (enhanced PRT model) on social responsiveness and expressive language in young autistic children.	PRISM enhaced PRT model (NDBI)	N.S.	Clinician delivered + parent mediated (mixed)	N.S.	6 months (10 hrs/week)	N.S.
**4**	To evaluate the effectiveness of a school-based peer social intervention aimed at increasing social engagement in school-age dyads including MV children.	Manualized, school-based peer social intervention (conversation and collaboration protocols)	special education schools	Clinician/Educator delivered	60	15 weeks (4session/week)	60
**5**	To assess changes in functional and symbolic play after a 6-month JASPER intervention and examine their relationship with expressive language in school-aged MV autistic children.	Naturalistic developmental behavioral intervention (JASP-EMT), with or without a SGD; focused on JASP, and language through	structured clinical environment	Clinician delivered	approx. 47–52 sessions total (dipende se early o slow)	6 months (2–3 sessions/week depending on treatment response<br>•Phase 1: 2s/w<br>•Phase 2: 2 or 3s/w)	60

		play-based sessions.					
**6**	To evaluate whether AMMT (intonation-based therapy) improves spoken language (syllables, consonants, vowels) in minimally verbal children compared to SRT.	AMMT: Intonation-based therapy using sung words and bimanual drumming to engage auditory–motor circuits.<br><br>SRT: Standard speech repetition without intonation or drumming, using the same stimuli.	N.S.	Clinician delivered	25	N.S.	N.S.
**7**	To examine whether orthographic support helps nonspeaking and MV autistic children learn new words, and which factors (e.g., language, reading skills, autism severity) influence this effect.	Computer-based mutual exclusivity word learning task with and without orthographic support	Remote / Home-based (computer-based task completed at home or school)	Clinician/research + parent mediated (mixed)	1	N.A.	29

**8**	To assess whether a brief JASPER intervention improves joint attention, symbolic play, and social communication in MV preschool autistic children who did not respond to prior intensive interventions.	Developmental, play-based social communication intervention (JASPER)	Non-public preschool specialized for autistic children	Clinician delivered	24	12 weeks (2sessions/week)	30
**9**	To evaluate a brief multi-component intervention (caregiver training, DTT, JASP+EMT+SGD) on social communication in young MVchildren and its maintenance after 4 months.	Combined DTT, JASP + EMT + SGD, and parent-mediated training	Intervention delivered in both clinic (2x/week) and home (1x/week) settings	Parent mediated	36 sessions total: 24 clinic-based and 12 home-based	4 months (3 sessions/week; 2 clinic, 1 home)	Clinic: 40–60 min; Home: 75–90 min per session
**10**	To assess the effectiveness of expert training and consultancy for teachers of autistic	PECS	School setting	Educator delivered	N.S.	5 months (N.S.)	N.S.

	children in the use of the PECS.						
**11**	To evaluate whether an adaptive intervention (JASP+EMT), with or without SGD, improves spontaneous spoken language in MVschool-aged autistic children.	Adaptive, blended developmental-behavioral communication intervention (JASP+EMT), with or without a SGD	University clinic playrooms at three research sites (UCLA, Vanderbilt University, Kennedy Krieger Institute)	Clinician + Parent mediated (mixed)	48 (± intensified to 60 for slow responders)	24 weeks (2 sessions/week; increased to 3sessions/week for slow responders in Stage 2)	60
**12**	To compare DTT and JASPER in improving spoken language and social communication in MVautistic preschoolers with developmental delay, and identify predictors of treatment response..	DTT and JASPER	Community preschool programs	Clinician delivered	108 (100 child + 8 caregiver)	6 months (5 sessions/week months 1–4; 3 sessions/week month 5; 2 sessions/week month 6)	60
**13**	To compare RMIA and MCT in promoting functional speech in MVautistic preschoolers and	RMIA: Structured DTT with motor imitation to elicit speech.	Individual clinic-based setting	Clinician delivered	36	12 weeks (3sessions/week)	45

	examine how child characteristics influence outcomes.	MCT: Naturalistic, play-based approach to promote spontaneous speech.<br><br>Both included parent responsiveness training.					
**14**	To compare the efficacy of MBCT versus traditional speech-language therapy in eliciting speech in nonverbal autistic children	MBC: music-based speech therapy using standardized melodies and rhythmic clapping.<br><br>Comparator: traditional speech and language therapy.	University clinic setting	Clinician delivered	20	5 weeks (4sessions/week)	45
**15**	To compare the effectiveness of PRT and the PECS in promoting spoken language development	Naturalistic behavioral interventions: PRT and PECS	In-home sessions and clinic-based	Clinician delivered + Parent	129	23 weeks (Initially 7 sessions/week, 5 in-home, 2 clinic-based, reduced to	120

	in MV young autistic children.		parent education	mediated (mixed)		3 sessions/ week in the final 8 weeks)	
**16**	To evaluate whether FPI improves parental responsiveness and enhances children's expressive language, especially in those with low initial language skills	FPI	Home setting	Parent mediated	12	12 weeks (1 sessions/week)	90
**17**	To examine whether adding a peer-mediated approach to an SGD intervention improves communication, reciprocity, and social participation in autistic preschoolers.	Peer-mediated SGD intervention with iPad voice-output apps	Natural preschool settings including classrooms, hallways, and adjacent rooms	Educator delivered	N.S.	9-19 weeks (2-3 sessions/week)	15
**18**	To examine how different language training methods and verbal imitation ability affect spontaneous spoken	Speech Alone, Sign Alone, Simultaneous Presentation of Sign and Speech, Alternating	N.S.	Clinician delivered	90	N.S.	40

	language in MVautistic children..	Presentation of Sign and Speech					

AMMT:Auditory-Motor Mapping Training; DDT:Discrete Trial Teaching; EMT:Enhanced Milieu Teaching; FPI:Focused Playtime Intervention; JASP:JASPER:Joint Attention, Symbolic Play, Engagement and Regulation; MBCT:Melodic-Based Communication Therapy; MCT:Milieu Communication Training; NDBI:Naturalistic Developmental Behavioral Interventions; PECS:Picture Exchange Communication System; PRISM: Pivotal Response Intervention for Social Motivation; PRT:Pivotal Response Treatment; RMIA:Rapid Motor Imitation Antecedent; SGDs:Speech-Generating Devices; SRT:Speech Repetition Therapy

Table S5. Domain-by-domain RoB assessment for the included randomised controlled trials, with corresponding AHRQ overall study quality.

Author	Random Sequence Generation	Allocation Concealment	Selective Reporting	Other Bias	Blinding of Participants and Personnel	Blinding of Outcome Assessment	Incomplete Outcome Data	AHRQ Standard
Kasari, 2023	Unclear	Unclear	High	Unclear	Low	Low	Low	Poor
Chenausky, 2022	Unclear	Unclear	Low	Low	Low	Low	Low	Fair
Barrett, 2020	Low	Low	High	Unclear	Low	Low	Low	Fair
Hampton, 2020	Low	Low	Low	Low	Low	Low	Low	Good
Bauminger-Zviely, 2019	Low	Unclear	High	Unclear	Low	Unclear	Low	Poor
Chang, 2018	Unclear	Low	High	Unclear	Low	Low	Low	Poor
Thiemann-Bourque, 2018	Low	Low	Low	Low	Low	Low	Low	Good
Almirall, 2016	Low	Low	Low	Low	Unclear	Unclear	Low	Fair
Kasari, 2014	Unclear	Low	Low	Unclear	Low	Low	Low	Fair

Schreibman, 2014	Low	Low	Low	Low	Low	Low	Low	Good
Goods, 2013	Low	Low	Unclear	Unclear	Low	Low	Low	Fair
Sandiford, 2013	Unclear	Unclear	Unclear	Unclear	Low	Unclear	Low	Poor
Siller, 2013	Unclear	Unclear	High	Low	Low	Unclear	High	Poor
Howlin, 2007	Low	Unclear	High	High	High	Unclear	High	Poor
Yoder, 1988	Unclear	Unclear	Unclear	Unclear	Low	Unclear	Low	Poor

Table S6. ROBINS-I risk-of-bias assessment for included non-randomised studies, with AHRQ overall study quality.

Study	Confounding	Selection of participants into the study	Classification of intervention	Deviations from intended interventions	Missing data	Measurement of outcomes	Selection of reported results	Overall
Abdi, 2023	Critical	Serious	Low	Serious	Low	Serious	Serious	Poor
Clark, 2022	Moderate	Moderate	Low	Low	Low	Low	Moderate	Moderate
Paul, 2013	Serious	Serious	Low	Moderate	Moderate	Moderate	Moderate	Poor
